# Artificial Determination of Sex

**Published:** 1898-02

**Authors:** 


### ARTIFICIAL DETERMINATION OF SEX.

There is nothing so fatal to medical progress as newspaper sensationalism. Science is a plant that can not be subjected to hothouse methods of stimulation during its growth without the most disastrous results.

When medical discoveries are unfortunate enough to come under the eye of newspaper reporters, and to be published with startling headlines, nothing but harm ever results. It was thus with tuberculin. It was thus with glandular extracts. It is sure to be thus with whatever the newspapers touch within this domain. The scatterbrained are stimulated to imitate experts and thus bring discredit upon work that as far as it has gone may be unimpeachable. Fools are still willing to rush in where angels fear to tread. A chance is given the advertiser to evade the code of medical ethics and get an abundance of free notices. Quacks and pretenders are enabled to pose as discoverers and the more easily to delude the public. The average man can not judge between good and bad scientific work. The reporters, as part of the public, are not always prepared to tell the difference between the sound and the spurious. When the very best work of the best men is given them they rarely report it properly, and their failure to give the true statement, together with their misleading headlines, make the whole matter aggravatingly misleading. It is seldom safe to trust to anything pretending to be scientific, if the least technical, when it appears in the form of a report in a daily paper. Where the matter is such as any intelligent man might understand, of course it is different. There a critical survey of what is said is likely to give some idea of the subject.

The latest sensation of the hour is an interview with Professor Schenck, of the University of Vienna, regarding what is reported as his discovery of the cause of sex. Until Professor Schenck's sealed paper on this subject to the Vienna Imperial Academy of Science has been published, there is really little known on which a safe judgment can be based concerning his claims. The sum total of all he told the reporter is that he knows how to direct the development of the embryo so as to give it what he claims to be a proper number of red blood-cells to make it a male. He feeds the mother some suitable form of diet. What that diet is, how he gives it, or what precautions are pursued has not yet been told. He states that he can tell in advance, from an examination of the expectant mother's "products", what will be the sex of her child. Whether the products referred to are excretions or secretions, or both, the report does not state, nor does it give the slightest information of how the knowledge is obtained by such an examination. Professor Schenck informed the reporter that he could tell whether a given egg would produce a hen or a rooster. Why he mentioned this it is hard to understand, as the feeding of a sitting hen could surely have no such effect upon the coming bird as the feeding of a mammalian mother. The incongruity of this association but emphasizes the fact that it is very unsafe to hold that inferences made from what he is said to have said are in any sense his belief.

Professor Virchow has raised a question that we fear it will be a long time before Professor Schenck can meet satisfactorily if the reporter has at all fairly presented his words. When a mother has twins of unlike sex both have been subjected to conditions of nourishment nearly alike. Why, then, were they not both males? By no artificial methods capable of execution can we ever hope to treat any two embryos in different mothers any nearer alike than twins are treated in a common uterus. If the nutrition of the mother determines the sex of the child, then twins should always be of the same sex, for the mother has the same treatment while carrying both.

That nutrition has something to do with determining sex is a well-established fact, and was known long before Dr. Schenck's claimed discovery. There are many facts that indicate that an unfavorable diet allows a larger number of male germs to develop than female ones. This, however, is wholly opposed to the reported new theory of Dr.Schenck, which denies the existence of any predetermination of sex and claims to produce what ever sex may be desired.

The professor distinctly says, according to his reporter, that "all former theories and hypotheses have proven false. According to my discovery, the man has no influence whatever on the sex of the child. It all depends upon the woman." There is nothing particularly abstruse or technical about this statement and surely the reporter would not deliberately misrepresent him. How, then, does the professor account for the fact that after a war a larger proportion of males are born than females? Why is it that in Utah under polygamy there was an excess of males? Why is it that every condition that tends to make the energy of the man less than that of the woman gives an increase in males? If we view the problem as one of survival of the strongest under adverse circumstances, it is easy to see the meaning of such facts. If we change our standpoint and take it to mean only the quality of nutrition taken by the mother, it becomes inexplicable. Under all conditions of the case only the averages are altered and that but slightly.

Even Professor Schenck does not seem to claim to be able to make mothers bear daughters exclusively, or to raise largely the production of daughters. The whole tendency of generation is to produce an excess of females under normal conditions.

In spite of this normal tendency, anything that weakens the average men of a nation increases the number of males that are born. To maintain equilibrium, nature seems to sow more females and when there is no great struggle for nutrition reaps a majority of the same kind. Once, however, let there come a strain on male vigor and the normal trend is broken and a majority of males result.—*American Medico-Surgical Bulletin.*

